# Personal

**Published:** 1901-11

**Authors:** 


### PERSONAL.

DR. P. M. RIXEY, medical inspector United States navy, who for several years served as physician at the White House, and was on duty with the President and Mrs. McKinley during their recent visit to Buffalo, which terminated so sadly, will, it is announced, be appointed surgeon-general of the navy upon the expiration of the term of Surgeon-General Van Reypen. President Roosevelt thus delicately carries out the intention of the late President McKinley, in bestowing fitting recognition upon Dr. Rixey of his conspicuous ability as an officer and faithfulness as a physician.

DR. A. L. GIHON, medical-director United States navy (retired), stationed at New York, lately spent a week in Buffalo as the guest of Dr. and Mrs. William Warren Potter, of Franklin Street. Dr. Gihon took active part during the meeting of the American Public Health Association, held in this city September 16-20. He also paid several visits to the exposition, where his sons Albert and Clarence, who reside at Paris, are represented in the gallery of arts with paintings that have attracted attention.

DR. GEORGE W. BRUSH, of Brooklyn, Commander of the Medal of Honor Legion, attended the eleventh annual meeting of the order held in Buffalo, October 9 and 10, 1901, and presided at the sessions. Dr. Brush was formerly a state senator, in which capacity he rendered valuable service to the medical profession in relation to bills that came up for consideration during his term.

DR. H. W. LONGYEAR, of Detroit, Mich., announces that he has removed his office and residence from 698 Woodward Avenue. Office, Rooms 1 and 2 Grand Circus Building, 271 Woodward Avenue. Telephone, Main 1418. Hours: 8 to 9 a.m., 1.30 to 3 p.m. Residence, 462 Jefferson Avenue. Telephone, Main 4244.

---

DR. GEORGE RYERSON FOWLER, of Brooklyn, Dr. Maurice J. Lewi, of New York, Dr. Eugene Beach of Gloversville, and Dr. A. Walter Suiter, of Herkimer, members of the State Medical Examining and Licensing Board, were among the recent visitors to the exposition.

---

DR. MAURICE J. LEWI, of New York, secretary of the State Medical Examining Board, has removed from 102 W. 81st Street to 149 W. 85th Street. Hours: 8.30 to 10 in the morning; 5 to 7 in the evening. Telephone call : 2059 Riverside.

---

DR. VICERZ CZERNY, professor of surgery at the University of Heidelberg, who has recently visited this country, was the guest, September 20, 1901, of Dr. Roswell Park. Professor Czerny visited the University of Buffalo and the exposition.

---

DR. CARL G. LEO-WOLF, of Niagara Falls, has removed his office to his residence, 1136 Main Street, corner Chilton Avenue. Hours: 9 to 10 a.m., 2 to 4 and 7.30 to 8.30 p.m. Telephone, Bell 244; Home 254.

---

DR A. M. CARTLEDGE, of Louisville, Dr. Edwin Walker, of Evansville, Dr. Joseph Price, of Philadelphia, and Dr. W. G. Macdonald, of Albany, have recently paid short visits to Buffalo and the exposition.

---

DR. AND MRS. JOHN E. BACON, formerly of Buffalo, now of Tombstone, Ariz., have recently paid a visit to Mr. and Mrs. John D. James, of Elmira, N. Y.

DR. AND MRS. WILLIAM D. HAGGARD, JR., of Nashville, were among the late summer visitors to Buffalo and the exposition. They expressed great delight with the city and the fair.

---

MR. FRANK A. RUF, of St. Louis, president of the Antikamnia Chemical Company, has been elected vice-president of the Fourth National Bank of that city.

Dr. A. A. Hubbell, of Buffalo, was elected president of the State Medical Association at its late annual meeting held in New York City.

---

Dr. M. F. Clausius, a graduate of the University of Buffalo, class of 1874, recently paid a visit to Buffalo and the Exposition. Dr. Clausius was appointed to medical service in the army last year, and has been serving in the Philippines since August 1900. He has been granted leave of absence for a month, on the expiration of which he is assigned to duty at Fort Grant, Arizona. Dr. Clausius had a varied experience in the department of Northern Luzon, P. I., and gives a favorable account of the progress there making toward a better civilisation.

